# Do Physicians’ Attitudes towards Patient-Centered Communication Promote Physicians’ Intention and Behavior of Involving Patients in Medical Decisions?

**DOI:** 10.3390/ijerph17176393

**Published:** 2020-09-02

**Authors:** Dan Wang, Chenxi Liu, Xinping Zhang

**Affiliations:** School of Medical Management and Health Management, Tongji Medical College of Huazhong University of Science and Technology, Wuhan 430030, China; 2019511009@hust.edu.cn (D.W.); liu_chenxi@hust.edu.cn (C.L.)

**Keywords:** patient-centered communication, patient involvement, primary care, Chinese revised-patient-practitioner orientation scale

## Abstract

Promoting patient-centered communication among physicians is one core strategy for improving physician–patient relationships and patient outcomes. Our study aims to understand the physicians’ attitudes towards patient-centered communication and its effects on physicians’ intention and behavior of involving patients in medical decisions in primary care in China. One cross-sectional study was conducted in primary facilities in Hubei province, China, from December 2019 to January 2020, where physicians’ attitudes towards patient-centered communication were measured by the Chinese-revised patient–practitioner orientation scale. Multilevel ordinal logistic regression was conducted for estimating the effects of physicians’ attitudes on their intention and behavior of patient involvement in medical decisions. Six hundred and seventeen physicians were investigated for the main study. Physicians had a medium score of patient-centered communication (3.78, SD = 0.56), with relatively high caring subscale score (4.59, SD = 0.64), and low sharing subscale score (3.09, SD = 0.75). After controlling physicians’ covariates, physicians’ attitudes towards patient-centered communication was significantly associated with a higher intention of involving patients in medical decisions (OR > 1, *p* = 0.020). Physicians’ positive attitudes towards patient-centered communication affected their intention of involving patients in medical decisions, which implies the importance of taking the physicians’ attitudes into account for the accomplishment of patient involvement processes.

## 1. Introduction

Patient-centered care is widely endorsed as a central component of high-quality health care [1,2]. In contrast to a paternalistic biomedical approach, The Institute of Medicine defines patient-centered care as “providing care that is respectful of and responsive to individual patient preferences, needs, and values and ensuring that patient values guide all clinical decisions” [3].

Patient-centered communication has been raised as one of a physicians’ core competencies and skills. It enables physicians to offer care that is concordant with the patient’s values, needs and preferences, as well as the patient’s involvement in making decisions about their care [4,5,6]. There is ample evidence of the importance of physician–patient communication, and patient-centered communication interventions have shown to improve the physician–patient relationship and patient healthcare outcomes [7,8]. Promoting patient-centered communication skills among physicians has been one recommended strategy by health scholars, health administrators, as well as healthcare organizations [4,8,9].

Numerous instruments have been developed to measure physicians’ patient-centered communication attitudes [10,11]. Originally developed by Krupat et al., the patient–practitioner orientation scale (PPOS) is one widely accepted instrument that was developed to assess physicians’, medical students’, and patients’ attitudes of patient-centered communication [12]. In China, Ting et al. and Wang et al. first attempted to use the patient–practitioner orientation scale (PPOS) to investigate patients’ and healthcare providers’ perceptions of patient-centered communication, and Wang et al. validated the revised PPOS in Chinese Context [13,14]. However, only one study was conducted in physicians with a small sample size of 116 with convenient sampling [13].

There was substantial evidence that physicians’ beliefs and attitudes were demonstrated as significant physician-related factors influencing patient participation in medical decisions [15,16]. However, the relationship between physicians’ attitudes of patient-centered communication and patient involvement in medical decisions was rarely investigated, and the previous researches mainly focused on general patient samples with the potential bias of heterogeneity regarding diagnosis and illness duration [17].

Acute respiratory infections (ARIs) are one of the most common reasons for visiting primary care. For most ARI cases, whether to treat patients with antibiotics or not is nearly at equipoise. Involving patients in medical decisions is especially appropriate in such cases that more than one clinically reasonable treatment option is available [18]. Therefore, we focused our study on specific patients with acute respiratory infections.

Our study aims: (1) To assess physicians’ attitudes of patient-centered communication; and (2) to explore whether the physicians’ attitudes of patient-centered communication was associated with physicians’ intention and behavior of involving patients in medical decisions for specific patients with ARIs. Specifically, we hypothesized that primary care physicians were patient-centered towards physician–patient communication; and more positive attitudes would increase physicians’ intention and their actual behavior of involving patients in medical decisions with patients with ARIs.

## 2. Materials and Methods

### 2.1. Study Setting

A cross-sectional study was conducted in Hubei, a province in central China with a population of 59.17 million with the middle social and economic development of all the regions [19]. The present study focused on primary care facilities covering urban community health centers (CHCs) and rural township health centers (THCs). According to the latest statistics, primary care facilities in Hubei served 43.53 million outpatients in 2018 [19].

### 2.2. Sampling and Participants

A multi-stage cluster sampling was adopted in this study. The first stage involved five cities from the 16 cities in Hubei province: The provincial capital of Hubei, Wuhan, and a random selection of other four prefecture-level cities. In the second stage, one urban district and one rural county were randomly selected from the included five cities, respectively. All the primary care facilities in each selected district/county were investigated in this study from December 2019 to January 2020. The detailed information on the geographic distribution of the participants was presented in Supplementary file 1 (Figure S1).

In this study setting of cases with ARIs, the following criteria for physicians were adopted: (1) Being able to prescribe antibiotics independently; (2) having authorized ≥100 prescriptions over the past three months prior to the survey to ensure a reliable estimation. Before the surveys, a list of physicians meeting the criteria in each selected primary care facility will be sent to the research team. The lists were provided by the local healthcare institutions based on the physicians’ profile and prescription data between January 2018 and December 2018.

### 2.3. Measurements

- Chinese Revised Physician-Practitioner Orientation Scale

The original PPOS was adapted to the Chinese context for measuring an individual’s attitudes towards the physician–patient relationship. The Chinese-revised patient–practitioner orientation scale (CR-PPOS) contains has been validated in the Chinese context with good reliability, validity, and discriminative power [13].

The CR-PPOS scale is a self-administered questionnaire, and it contains 11 items reflecting two domains subscales of caring and sharing. Of the 11 items, five items were used to evaluate caring attitudes assessing the extent to which the physician believes they should care about patients’ emotional needs and regard the patient as a whole person. The remaining six items were used to evaluate sharing attitudes, which physicians perceive they are oriented to share power with patients in medical decisions. Scoring was based on a 6-point Likert from strongly disagree = 1 to strongly agree = 6. The CR-PPOS score is inverted so that higher caring and sharing scores representing more patient-centered inclination. The average maximum score and minimum score was 6 points and 1 point for the CR-PPOS items, with 3.5 as the cutoff point for judging the physicians’ attitudes. The CR-PPOS instrument was presented in supplementary materials (Table S1).

- Outcome measures

Outcome 1: Physicians’ intention of patient involvement in medical decisions was measured through five scenarios based on the Control Preference Scale. Physicians were provided with the five scenarios with patients with ARIs. They are asked to choose one scenario which best exemplifies their preferred way in clinical practice. Physicians’ intention was divided into three categories according to the extent to which physicians were likely to share the power with patients: Paternalistic, informative, and shared decision-making. The specific information of the scenarios was provided in Table 1.

Outcome 2: Physicians’ behavior of patient involvement was measured by the self-reported actual proportion of involving patients in medical decisions. The responses were scored from 0% to 100% under the context of cases with ARIs. This outcome measure was divided into three categories by three levels of patient involvement (low level: 0~30%; middle level: 40~60%; high level: 70~100%).

### 2.4. Data Collection

A total list of 779 physicians was provided before the surveys. The research team was trained to contact the physicians on the list by one to one approach to ensure that physicians completed the questionnaire independently. Physicians who were on vacation or were retired from the facility were excluded from this study.

With oral consent, participants were asked to complete a questionnaire in a paper form containing the CR-PPOS and outcome measures. In addition, demographic information (gender, age, educational level, years of practice, professional title, etc.) were also collected. The data source was presented as Data S1 in supplementary files.

It is worthy to note the specific situation of medical education in China. The current law (the Law for Licensing Medical Practitioner) allows medical graduates with a non-bachelor degree to become assistant doctors. After years of working experience, and if they pass an examination, they will have the chance to obtain the full doctor licensure [20]. Therefore, it is possible for physicians with an education level below college degree.

### 2.5. Ethics

The study was approved by the Ethics Committee of Tongji Medical College, Huazhong University of Science and Technology (IORG: IORG 0003571).

### 2.6. Statistical Analysis

The *t*-test and analysis of variance (ANOVA) for parameter testing and the Mann-Whitney U test or Kruskal–Wails test for non-parameter testing were conducted for comparing the average scores of overall CR-PPOS, sharing subscale, and caring subscale among different physician groups.

Cronbach’s α factor and confirmatory factor analyses to determine the reliability and validity of CR-PPOS. The previously validated PPOS scale yielded Cronbach coefficient alpha values of 0.68, which is higher than an acceptable value of 0.6 for an instrument with a relatively small number of items. The confirmatory analysis supported the discriminant two factors of the PPOS scale with acceptable model fit indices with CFI values, and TLI values were over 0.95, and RMSEA was less than 0.60 [21].

Prior to the multilevel analyses, the comparisons of average scores of overall CR-PPOS, sharing subscale, and caring subscale were also conducted among different groups of physicians’ intention (paternalistic, informative, and shared decision-making) and actual proportion of patient involvement (high level, middle level, and low level) by ANOVA test or Kruskal–Wails test.

Since it was speculated that physicians in the same institution had similar attitudes of patient-centered communication, multilevel ordinal logistic regression analyses were applied with questionnaire data at physician level nested in clusters (physician’s affiliated primary care facility). Physicians’ intention of paternalistic, informative, and shared decision-making ways and three levels of the actual proportion of involving patients in medical decisions were set as ordinal dependent variables. The multilevel analyses provides interpretable parameters of odds ratio (OR), which corresponds to the effects of study variables on the probability of the dependent variables.

Physicians’ demographic characteristics were also included as covariates in the analyses. Only cases with complete data were included in the analysis. The statistical analyses were performed using STATA (version 12.0, StataCorp, Texas, TX, USA) and Mplus (version 6.0, Muthén & Muthén, Los Angeles, CA, USA). Statistical significance was set at *p* < 0.05.

## 3. Results

### 3.1. Physicians’ Demographics and Attitudes of Patient-Centered Communication

A total of 617 physicians from 102 primary care facilities were investigated in the main study. Among the 617 physicians, 63.0% were male, and 37.0% were female. The majority of physicians have a college or graduate degree (83.1%). Physicians had an average of 16 years of working practice, and over 80% of physicians received antibiotic training last year. Over 70% of the respondents were from general practice (49.59%) and internal medicine (21.39%) (Table 2).

The physicians received an average CR-PPOS score of 3.78 (SD = 0.56), with an average score of 4.59 (SD = 0.64) of the caring subscale and 3.09 of the sharing subscale (SD = 0.75) (scores higher than 3.5 indicate patient-centered attitudes). The scores of caring and sharing were compared among different physician groups. For example, female physicians had slightly higher caring (4.64 vs. 4.58) and sharing (3.14 vs. 3.09) scores than male physicians, although the differences were not statistically significant (Supplementary file 3: Figure S2). Except that physicians in the younger age group and receiving antibiotics training group showed significantly higher scores of caring (*p* < 0.05), the caring and sharing scores between different physician groups were not statistically different. Physicians’ demographic characteristics and CR-PPOS scores were presented in Table 2.

### 3.2. Comparisons of CR-PPOS Scores among Groups Based on Physicians’ Intention and Behavior of Involving Patients in Medical Decisions

**Figure 1:**
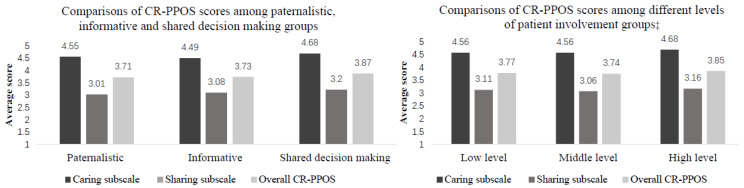
Comparisons of CR-PPOS scores among physician groups, based on intention and behavior of patient involvement. Note: The way of variance (ANOVA) was conducted for parameter testing and Kruskal-Wails test for non-parameter testing. Scores higher than 3.5 indicate positive attitude. ‡ represents 26 cases were missing with incomplete data of study variables.

The descriptive results of physicians’ intention and behavior of involving patients in medical decisions, and comparisons of CR-PPOS scores among different groups based on intention and behavior of patient involvement were presented in Figure 1.

Of the total 617 physicians, the majority of physicians preferred paternalistic (*n* = 241) or shared decision way (*n* = 286) of medical decisions in primary care. Compared with the paternalistic way of decision-process, physicians had significantly higher scores of caring, sharing, and overall CR-PPOS (*p* < 0.05). Physicians with the intention of shared decision-making had a higher CR-PPOS score than physicians in an informative way (3.87 vs. 3.73) and paternalistic way (3.87 vs. 3.71).

There were over 60% of physicians with the middle or high level of the proportion of involving patients in medical decisions (low level: *n* = 213; middle level: *n* = 138; high level: *n* = 240). Although physicians with a higher proportion of involving patients in medical decisions showed higher scores of CR-PPOS scores, however, the associations between practical behavior and attitudes were not significant (*p* ≥ 0.05). The detailed information of associations between physicians’ attitudes of patient-centered communication and intention and behavior of involving patients in medical decisions was presented in Table 3.

### 3.3. Relationship between Physicians’ Attitude of Patient-Centered Communication and Intention and Behavior of Involving Patients in Medical Decisions

The multilevel ordinal logistic regression was conducted to explore the relationship between CR-PPOS and intention of patient involvement (paternalistic, informative, and shared decision-making) and the actual level of patient involvement (high level, middle level, and low level) after adjusting the confounders.

The sharing dimension was significantly associated with physicians’ intention of engaging patients in medical decisions (OR = 1.05, *p* = 0.020), indicating every point increase of sharing score would contribute to a 5% increase in physicians’ intention of sharing power with patients with ARIs in medical decisions. Younger physicians (age ≥ 35 and < 50 years: OR = 0.51, *p* = 0.023; age ≥ 50 years: OR = 0.24, *p* < 0.001) with higher educational level (vocational education: OR = 1.87, *p* = 0.014; college education and above: OR = 1.65, *p* = 0.077) showed higher level intention of engaging patients with ARIs in medical decisions.

On the other hand, physicians’ positive attitudes towards caring and sharing dimensions had slight effects on the practical level of patient involvement with patients presenting with ARIs, although the effects were not statistically significant (*p* > 0.05). Year of practice was found as one significant predictor of a higher level of patient involvement (OR = 1.02, *p* = 0.049). In addition, physicians in THCs were less likely to prefer or actively engage patients in medical decisions with patients with ARIs (OR = 0.54, *p* = 0.009). The detailed information of multilevel analysis results was presented in Table 3.

## 4. Discussion

Appling the 11-item CR-PPOS instrument, physicians showed a positive attitude of patient-centered communication, with relatively high ratings of the caring subscale and low ratings of the sharing subscale. The multilevel ordinal logistic regression analyses showed that patient-centered sharing was significantly associated with physicians’ intention of involving patients in medical decisions, while physicians’ attitudes did not affect their actual level of patient involvement.

The findings related to PPOS scores were essentially consistent with previous Chinese-based and studies based in some Eastern and Western countries [22,23,24,25,26]. The findings reported that physicians rated relatively high caring and low sharing scores, which indicates a similar pattern that physicians were more patient-centered in the caring subscale than in the sharing subscale [22,23,24,25]. However, the differences between caring and sharing scores were much higher than the results reported by other researches, with much less patient involvement in information and decision-making. Specifically, the sharing score of our study (3.09) is much lower than the results of Japan (4.26~4.39), Korean (3.61), and Brazil (4.10) [22,25,26].

The observed lower score of sharing subscale among Chinese physicians in primary care may contribute to environmental factors, such as Chinese cultural values, local health system [27].

Compared with the western cultural values of highlighting individual autonomy, the collectivistic nature of Chinese culture often leads to a decision-making process where the individual’s preferences are informed by others or secondary to others’ preferences [28]. In addition, the previously widely demonstrated drawbacks of the primary care system in China, such as lack of resources (e.g., shared decision-making training, decision aids systems) and lack of trust, may be the potential reasons responsible for the low scoring of sharing power with patients in medical decisions [29]. Furthermore, it was important to mention that previous studies conducted in China and other countries were on general patients (and did not specify a illness the patient was suffering from); therefore, the specific choice of ARI cases may be one potential factor influencing the caring and sharing scores, and should be further explored in future studies.

We found that physicians’ positive attitudes about sharing affected physicians’ intention to involve patients in medical decisions [15,30]. Although these differences were small on the metric of the scales in this study, they were translated to have moderate to large effect sizes on physicians’ intention. Based on social cognitive theories, such as the theory of planned behavior, several researchers have demonstrated a positive attitude was one significant predictor of the behavior intention of the shared decision process with patients in medical decisions [15,30]. One recent study conducted by Drivenes reported more positive attitudes of patient-centered communication were associated with patient perception of the shared decision-making process in mental health [17]. It was expected that higher degrees to which physicians believe they should share decision-making information and power with the patients contributed to a higher intention of conducting a shared decision-making approach in primary care.

However, in particular, physicians’ attitudes of patient-centered communication failed predicted actual behavior of engaging patients in medical decisions. The potential reasons were presented, as follows.

As the decision-making process is the interpersonal process of both physicians and patients, both physicians and patients would be a fundamental unit of achieving patient-centered communication [17]. The implementation of involving patients in medical decisions is a challenge, due to the patients’ condition in the present study. Ting and Wang et al. reported that Chinese patients would prefer to rely on physicians in medical encounters, and one reported barrier by Chinses patients of patient-centered care was that they were incapable of clearly making up an opinion of what matters most and able to express it due to limited medical literacy [13,14]. The incapability or unwillingness to participate in medical decisions would impede, at least in part, physicians’ actual behavior of involving patients in medical decisions. Moreover, the contextual factors, such as time constraints, would also negatively affect physicians’ actual behavior of involving patients in medical decisions [31,32].

As for the effects of covariates, our findings mirror previous researches in which younger physicians with higher educational levels had a higher tendency to involve patients in medical decisions. Training physicians have been identified as one core strategy for implementing a shared decision process, and there is some evidence to suggest that younger primary care physicians were more likely to engage more in partnership with patients and more involved in medical encounters [33,34].

Identified as one significant predictor of the actual level of patient involvement, increasing years of practice would contribute to accumulated knowledge and expertise, which may serve as the basis for collaboratively weighing the treatment options and sharing power with patients [35].

The facility type of primary care facility was also demonstrated as one important predictor of physicians’ intention and their actual behavior of involving patients in medical decisions. It was likely that physicians in THCs would be more likely to face patients of low literacy, and would serve as the delegates of patients as “decision-makers” [36]. Moreover, the physicians in THCs would have fewer opportunities and resources of patient-centered communication skills [37], which would lead to failure of expressing a friendly attitude towards patients or involving them in decision-making.

### 4.1. Study Implications

The study also provided some implications for practice and future research. Since physicians with more positive attitudes towards patient-centered communication had a higher intention of involving patients in medical decisions and physicians’ intention was statistically associated with physicians’ practical behavior, there may be implications for administrators and healthcare providers to emphasis the importance for education and training that equips and empowers physicians to apply patient-centered communication in medical decisions, especially in rural facilities in primary care. The measures, such as making patient-centered communication as education curricula of medical students or as part of clinical guidelines, providing decision-making aids tools, would be potential strategies of training physicians in primary care [38,39,40]. Further research has to examine whether such strategies indeed might help to promote patient-centered communication of physicians, and as a result, improving the behavior of involving patients in medical decisions.

### 4.2. Strengths and Limitations

A strength of this study is that we considered the nested structure of physicians’ questionnaire data in the physician’s affiliated primary care facility by performing a multilevel ordinal logistic regression analysis. Physicians working in the same facility may share similar attitudes towards patient-centered communication. In addition, the measurements of CR-PPOS and outcome measures focused on specific patients in specific consultations with ARIs, which would help control the heterogeneity bias caused by a general sample of different disease burdens or illness duration.

There were also several limitations to our study. Firstly, since this was one cross-sectional study, the causal relationships between physicians’ patient-centered communication attitudes and physicians’ intention should be explained with caution. The finding was derived from one central province in China, which may limit the generalizability of our findings. At least, however, we conducted a large study in primary care with multi-stage cluster sampling, physicians nested in 102 primary care facilities from both rural and urban facilities were analyzed. Secondly, it is widely acknowledged that physicians’ self-assessment does not necessarily reflect their actual competence [41].

## 5. Conclusions

In the present study, physicians hold the average 3.78 overall CR-PPOS scores (higher than 3.5). This indicates relative positive attitudes towards patient-centered communication. Specifically, they had higher average scores on the caring subscale (4.59), and much lower scores of the sharing subscale (3.05). Physicians with positive attitudes towards the sharing subscale significantly increased their intention of involving patients in medical decisions. However, it is interesting to note that the attitude of the physicians regarding patient-centered communication did not impact their actual behavior of involving patients in medical decisions.

## Figures and Tables

**Table 1:** Physicians’ intention of involving patients in medical decisions with patients with acute respiratory infections (ARIs) based on Control Preference Scale.

**Paternalistic Way**
The physician makes the final medical decisions with the patient with ARIs, e.g., whether to uses antibiotics or not; The physician makes the final medical decisions, e.g., whether to uses antibiotics or not, with careful consideration of the opinions of the patient with ARIs.
**Informative Way**
The patient with ARIs makes the final decision of the antibiotic treatment, e.g., whether to uses antibiotics or not; The patient with ARIs makes the final decision of the antibiotic treatment, e.g., whether to uses antibiotics or not, with careful consideration of the physician’s opinions.
**Shared Decision-Making Way**
The physician and the patient with ARIs share the responsibility of the medical decisions, e.g., whether to uses antibiotics or not.

**Table 2:** Physicians’ demographic characteristics and associations between Chinese-revised patient–practitioner orientation scale (CR-PPOS) scores and physicians’ characteristics (*n* = 617).

Characteristics	*n* (%)/Media *n* (IQR)	Caring Subscale ^‡^	*p*-Value	Sharing Subscale ^‡^	*p*-Value	Overall CR-PPOS ^‡^	*p*-Value
		(>3.5, Good)		(>3.5, Good)		(>3.5, Good)	
Sex			0.584		0.506		0.444
Male	389 (63.00)	4.58 (0.67)		3.09 (0.78)		3.77 (0.57)	
Female	228 (37.00)	4.64 (0.61)		3.14 (0.73)		3.82 (0.54)	
Age (years)			0.001 *		0.301		0.301
<35	102 (17.03)	4.59 (0.66)		3.08 (0.74)		3.76 (0.55)	
≥35 and <50	355 (59.27)	4.68 (0.62)		3.14 (0.75)		3.84 (0.55)	
≥50	142 (23.27)	4.44 (0.67)		3.05 (0.76)		3.68 (0.51)	
Facility setting			0.325		0.260		0.219
Community health center (CHC)	220 (35.70)	4.59 (0.64)		3.07 (0.69)		3.76 (0.55)	
Township health center (THC)	397 (64.30)	4.61 (0.66)		3.13 (0.79)		3.80 (0.57)	
Specialties			0.190		0.940		0.319
General practice	306 (45.59)	4.59 (0.64)		3.11 (0.76)		3.82 (0.55)	
Internal medicine	132 (21.39)	4.56 (0.67)		3.09 (0.76)		3.86 (0.60)	
Surgery	70 (11.35)	4.56 (0.66)		3.10 (0.78)		3.85 (0.57)	
Others	109 (17.6)	4.74 (0.61)		3.14 (0.72)		3.80 (0.52)	
Educational level			0.313		0.975		0.631
Senior high school and below	104 (16.90)	4.54 (0.63)		3.11 (0.73)		3.76 (0.51)	
University degree	499 (80.80)	4.63 (0.65)		3.11 (0.77)		3.80 (0.57)	
Graduate	14 (2.30)	4.40 (0.81)		3.15 (0.78)		3.72 (0.70)	
Professional title ^†^			0.401		0.504		0.754
Resident physicians	302 (49.00)	4.53 (0.66)		3.12 (0.79)		3.80 (3.73)	
Attending physicians	234 (38.00)	4.60 (0.62)		3.08 (0.72)		3.77 (0.54)	
Associate or chef physicians	80 (13.00)	4.55 (0.69)		3.17 (0.88)		3.80 (0.61)	
Training regarding antibiotics			0.031 *		0.272		0.074
last year							
Yes	502 (81.40)	4.63 (0.65)		3.12 (0.77)		0.38 (0.56)	
No/don not know	115 (18.60)	4.50 (0.65)		3.04 (0.71)		3.71 (0.56)	

IQR, interquartile ranges (25th to 75th percentile). ^†^ represent there were missing cases; ^‡^ Maximum = 6, Minimum = 1, scores higher than 3.5 indicate more patient-centered communication. * *p* < 0.05.

**Table 3:** Results from multilevel ordinal logistic regression that examine the relationship between CR-PPOS and physicians’ intention and behavior of patient involvement.

	The Intention of Patient Involvement			The Behavior of Patient Involvement ^§^		
	OR ^†^	95% CI ^‡^	*p*-Value	OR ^†^	95% CI ^‡^	*p*-Value
CR-PPOS						
Caring subscale	1.01	0.95~1.06	0.652	1.04	0.98~1.09	0.14
Sharing subscale	1.05	1.01~1.09	0.020 *	1.01	0.98~1.05	0.574
Physicians’ age (Reference group < 35 years)						
≥35 years and<50 years	0.51	0.28~0.91	0.023 *	0.68	0.41~1.14	0.148
≥50 years	0.24	0.11~0.50	<0.001 *	0.53	0.27~1.12	0.1
Physicians’ gender (Reference group = male)	1.25	0.84~1.85	0.261	0.93	0.62~1.39	0.713
Years of practice	1.01	0.98~1.02	0.677	1.02	0.96~1.04	0.049 *
Specialties (Reference group: general practice)						
Internal medicine	0.7	0.46~1.05	0.09	0.8	0.53~1.21	0.303
Surgery	0.7	0.41~1.18	0.183	0.56	0.28~1.10	0.094
Others	0.91	0.57~1.44	0.71	1.13	0.51~2.53	0.75
Education level (Reference group = high school and below)	1.87	1.13~3.11	0.014 *	0.96	0.59~1.53	0.854
Vocational education	1.65	0.86~2.34	0.077	1.01	0.59~1.76	0.98
College education and above						
Receiving antibiotics training	1.3	0.84~1.89	0.262	1.49	0.98~2.28	0.06
Professional title (Reference group: resident physician)	0.99	0.65~1.48	0.949	0.85	0.57~1.26	0.435
Attending physician	1.03	0.54~1.97	0.913	0.82	0.42~1.59	0.56
Associate physician or chief physician						
Facility setting (Reference group: CHCs, community health centers)	0.75	0.50~1.13	0.178	0.54	0.34~0.82	0.009 *

^†^ OR, odds ratio, higher odds ratio showed a higher probability of physicians’ intention and behavior of involving patients in medical decisions; ^‡^ CI, confidence interval; ^§^ 26 missing cases with incomplete data of study variables * *p* < 0.05.

## Supplementary Materials

The following are available online at https://www.mdpi.com/1660-4601/17/17/6393/s1, Figure S1: The geographic distribution of the participants in Hubei Province, China; Figure S2: The distribution of CR-PPOS scores split by male and female; Table S1: Chinese Revised Practitioner-Patient Orientation Scale (English Version); The data source was presented as Data S1.txt.
