# Supplementary material for: Do Physicians’ Attitudes towards Patient-Centered Communication Promote Physicians’ Intention and Behavior of Involving Patients in Medical Decisions?
Source: Int J Environ Res Public Health. 2020 Sep 2;17(17):6393. doi: 10.3390/ijerph17176393 (PMC7503802; doi:10.3390/ijerph17176393)
Supplement: Supplementary file 1 [file ijerph-17-06393-s001.zip › Supplementary file2.docx]

**Supplementary file 2 Chinese Revised Practitioner-Patient Orientation Scale (English Version)**

| **Caring subscale** |
| --- |
| 1. When doctors ask a lot about personal backgrounds, they will be suspected of prying the privacy of patients.  *a. Strongly disagree b. Disagree c. Slightly disagree d. Slightly agree e. Agree f. Strongly agree* |
| 2. If doctors are truly good at diagnosis and treatment, the way of his/her communication with patients is not that important.  *a. Strongly disagree b. Disagree c. Slightly disagree d. Slightly agree e. Agree f. Strongly agree* |
| 3. If a doctor spends too much honesty and enthusiasm in the doctor-patient communication, he/she wouldn’t have made great achievements.  *a. Strongly disagree b. Disagree c. Slightly disagree d. Slightly agree e. Agree f. Strongly agree* |
| 4. Most patients in clinics want to leave the doctors’ office as quickly as possible (so as to reduce the time communicating with doctors)  *a. Strongly disagree b. Disagree c. Slightly disagree d. Slightly agree e. Agree f. Strongly agree* |
| 5. For doctors, knowing the patient’s culture and backgrounds is not very important for treating illness.  *a. Strongly disagree b. Disagree c. Slightly disagree d. Slightly agree e. Agree f. Strongly agree* |
| **Sharing subscale** |
| 6.The doctor is the one who should decide what gets talked about during a visit.  *a. Strongly disagree b. Disagree c. Slightly disagree d. Slightly agree e. Agree f. Strongly agree* |
| 7.Patients should rely on doctor’s knowledge and not try to find out their conditions on their own.  *a. Strongly disagree b. Disagree c. Slightly disagree d. Slightly agree e. Agree f. Strongly agree* |
| 8. Patients generally want reassurance rather than information about their health.  *a. Strongly disagree b. Disagree c. Slightly disagree d. Slightly agree e. Agree f. Strongly agree* |
| 9. If a patient does not agree with the opinions of a doctor, then it means that the doctor doesn’t get the patient’s respect and trust.  *a. Strongly disagree b. Disagree c. Slightly disagree d. Slightly agree e. Agree f. Strongly agree* |
| 10. The patient must always aware that the doctor is in charge.  *a. Strongly disagree b. Disagree c. Slightly disagree d. Slightly agree e. Agree f. Strongly agree* |
| 11. When patients look up medical information on their own, this usually confuses than it helps.  *a. Strongly disagree b. Disagree c. Slightly disagree d. Slightly agree e. Agree f. Strongly agree* |
