# Supplementary figures and images for: Do Physicians’ Attitudes towards Patient-Centered Communication Promote Physicians’ Intention and Behavior of Involving Patients in Medical Decisions?
Source: Int J Environ Res Public Health. 2020 Sep 2;17(17):6393. doi: 10.3390/ijerph17176393 (PMC7503802; doi:10.3390/ijerph17176393)

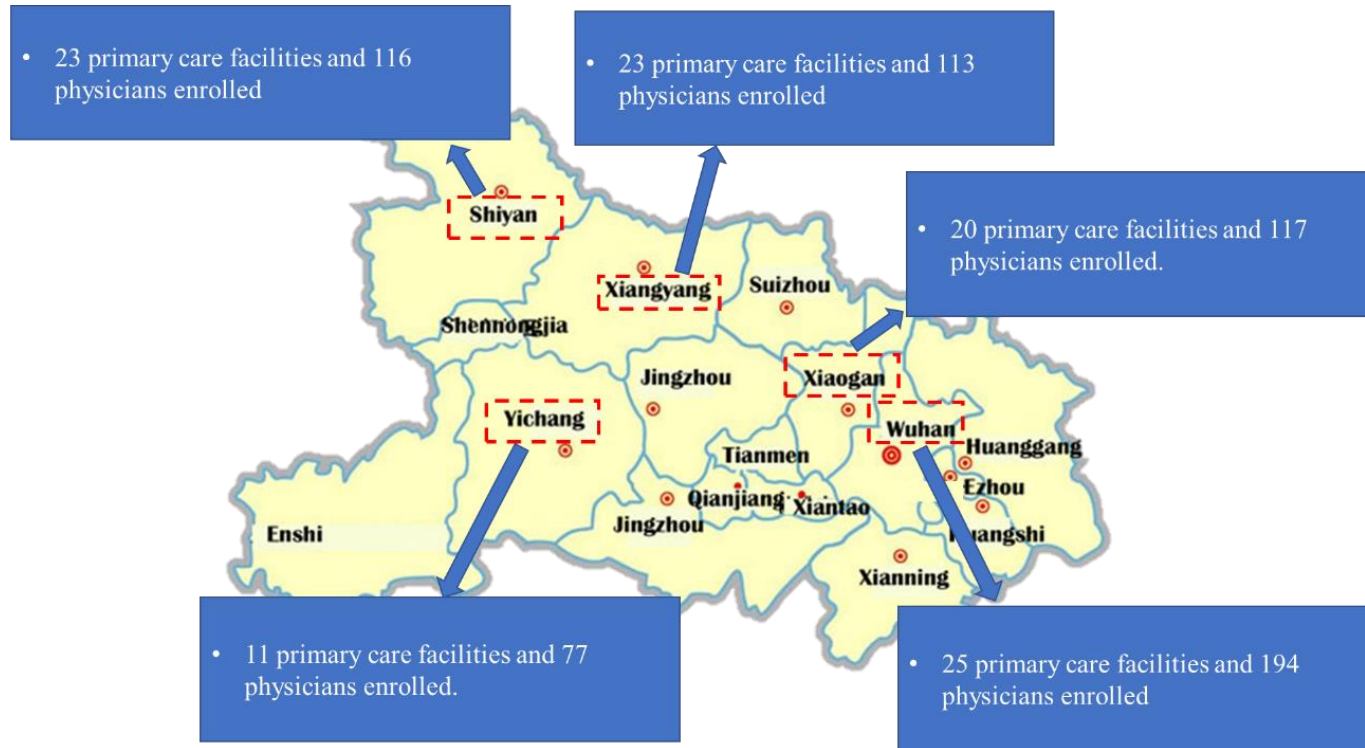

**Figure S1** The geographic distribution of the participants in Hubei Province, China

Supplement: Supplementary file 1 [file ijerph-17-06393-s001.zip › Supplementary file1.pdf]

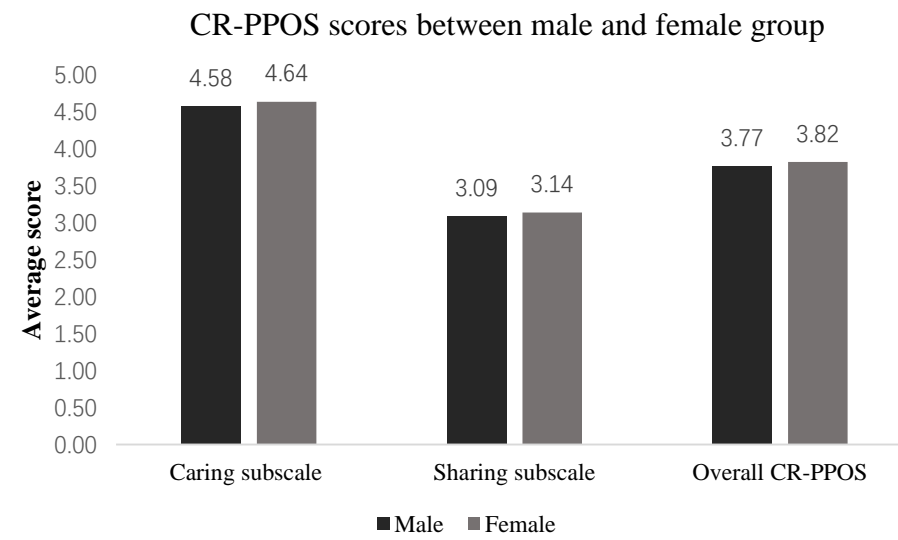

**Figure S2** Distribution of CR-PPOS scores split by male and female

Supplement: Supplementary file 1 [file ijerph-17-06393-s001.zip › Supplementary file3.pdf]
